# Evaluation of Attitudes toward Corporal Expression in Primary School Students: Validation and Psychometric Properties of a Questionnaire

**DOI:** 10.3390/ijerph20043463

**Published:** 2023-02-16

**Authors:** Jorge Rojo-Ramos, Irene Polo-Campos, Claudio Hernández-Mosqueira, Santiago Gomez-Paniagua

**Affiliations:** 1 Physical Activity for Education, Performance and Health, Faculty of Sport Sciences, University of Extremadura, 10003 Cáceres, Spain; 2 BioẼrgon Research Group, Faculty of Sport Sciences, University of Extremadura, 10003 Cáceres, Spain; 3 Departamento de Educación Física, Deportes y Recreación, Universidad de La Frontera, Temuco 4780000, Chile

**Keywords:** corporal expression, primary school, physical education, attitudes, validation, questionnaire

## Abstract

The content of corporal expression, usually forgotten by some teachers, has been shown to have favorable effects on the physical, social, and psychological health of students at all levels of education. Likewise, students’ attitudes should be positively influenced in the school environment in order to improve the teaching–learning process of the different contents taught. The purpose of this study was to provide the factor structure and validity of a questionnaire used to gauge pupils’ attitudes toward corporal expression. The sample consisted of 709 students in the last cycle of primary school from schools in the region of Extremadura (Spain). Reliability testing as well as confirmatory and exploratory factor analyses were conducted. The findings revealed a factor structure with three dimensions composed of 30 items with high reliability (Cronbach’s alpha = 0.85–0.90) and good and exceptional goodness-of-fit values. As a result, the questionnaire can be seen as a quick and simple instrument to use in analyzing students’ attitudes toward corporal expression and enabling stakeholders to take action to support it.

## 1. Introduction

Since the origin of humanity and the birth of each individual, corporal expression (CE) has existed. With the influences of Noverre and Delsarte and the emergence and development of the European Gymnastic Schools alongside the American influences, the world of the arts, psychology, pedagogy, and the social and cultural climate of the 1960s, it began and forged its development as a discipline in the middle of the 18th century [1]. It emerged from a culture that rejected the norms of daily life and restrained the body without any intellectual or ethical justification [2]. The General Law of Education [3] revealed its genesis and development within the Spanish legal system. The Organic Law on the General Organization of the Educational System [4] and later the Organic Law on Education [5] subsequently consolidated it as the curriculum for physical education (PE). Finally, after modifications of the Organic Law for the improvement of educational quality [6], it is being maintained to give greater freedom to the autonomous communities.

The progress of favorable characteristics for the integral development of the person, such as imagination, self-knowledge, and cooperative work within the group, can be adequately facilitated by CE, a particular component of the subject of PE [7]. A more updated definition would be Schinca’s definition [8], which states that it is a discipline that starts from the physical, connects with the internal processes of the person, and channels their expressive possibilities toward a creative gestural language; or that of Archilla and Perez [9] who stated that it is valued as a content of the integral formation of the person and favors great virtues for coexistence and life in society and cooperative and emotional living, although at the same time it is a content that generates insecurities and fears regarding difficulties in its planning and implementation in teachers. Thus, despite its advantages, which teachers consider crucial [10], a recent study revealed flaws in teachers’ instruction across the board. In primary and secondary education, they are associated, for instance, with a perception of low proficiency in the subject and its ensuing difficulty [11].

According to a study by Atkinson et al. [12], affect perception of the body is evaluated for both form and motion signals. The work by Omlor and Giese and its more thorough follow-up study [13] showed the existence of emotion-specific spatiotemporal motor primitives that underlie human gait using a systematic method. Body language has always considered the dual nature of space (physical and emotional) [14], which is more pronounced in children because it is a part of their affectivity and emotionality, which they inhabit, own, and symbolically modify [15]. The use of physical expression encourages children’s imagination and creativity while also enabling them to build or enhance all types of spatial conceptions, which serve as the foundation for later geometric development in primary and secondary school pupils [16]. However, when students encounter high amounts of emotional states such as irritation, anxiety, fear of failure, etc., they become demotivated [17]. While there is a clear need to create technologies that exploit the body as an effective communication modality, there is a less clear understanding of how these systems should be built, validated, and compared [18].

One of the barriers of CE is that it is a very limited work carried out in the classroom. Larraz [19] claimed that students make references to various sports and physical activities such as basketball, soccer, or athletics, but that there is no cultural support that ensures the success of these kinds of endeavors. Instead, students are frequently drawn to the various sports featured on television or in other media, and those that are concerned with body expression are less important [20]. On the other hand, one of the advantages of CE is that it can improve and help students’ development through culture. Valls and Padrol [21] argued that music and film are two concepts that are inextricably linked, which is a noteworthy justification for including music in further CE sessions while the cinematographic visualization is carried out. According to Martinez Rodrigo and González-Lucini [22], it is a potentially useful medium for the acquisition of values and positive behaviors. Additionally, Abad, Campos, Cortés, and Lienas [23] asserted that combining CE with cinema can ensure the activity’s success.

In this context, there are a variety of instruments that have been used to analyze aspects related to CE. For example, one of them assessed in a general way the importance given by the student to PE (with the content of CE appearing in only one of the 22 items) [24]. Two instruments [25] complemented the implementation of a training plan for CE, but their respective purposes were to check the variations generated by the implementation of the training plan in terms of student inclusion and to evaluate the work done by the students and the teacher as well as the quality of the training plan. For this reason, a scale was developed in 2020 to assess student attitudes about CE in the context of PE by unifying a number of previous tools that were less general but content-related, which resulted in a much more specific instrument [26]. Consequently, the objective of this study was to analyze the psychometric properties as well as the validity and reliability of a questionnaire aimed at assessing attitudes toward corporal expression in students in the last cycle of primary school in the Autonomous Community of Extremadura (Spain). In this way, we intended to check whether the tool was safe and reliable in determining the current status of students with respect to the content and thus the design actions aimed at improving attitudes toward CE.

## 2. Materials and Methods

### 2.1. Participants

The sample consisted of 709 students in the last cycle of primary school (from 10 to 12 years old) in Extremadura (Spain) from both public and private schools. Table 1 shows the sociodemographic characteristics of the participants, all of whom were selected on the basis of a convenience sampling method.

### 2.2. Instruments

A sociodemographic 6-item questionnaire that included questions on gender, age, center environment, type of center, course, and province of the school was developed to help describe the sample. 

Similarly, an instrument composed of 32 items divided into 4 different dimensions that was previously developed and validated in Spanish [26] was used to analyze students’ attitudes toward corporal expression. Dimension 1, “Evaluation of CE” (14 items), pointed to CE’s overall significance in life in general. Dimension 2, “Preference” (7 items), compared and contrasted the attitude toward CE versus other contents. Dimension 3, “Pleasure” (6 items), emphasized the student’s favorable experiences with CE. Finally, Dimension 4, “Teacher’s Attitude” (5 items), provided the student’s perception of the teacher’s facilitation or assistance in fostering their positive attitude or motivation for CE. Prior to the data analysis, the indirect questions were transposed to correspond to each of the aforementioned criteria. In addition, the responses were based on a Likert scale of 1 (strongly disagree) to 5 (strongly agree). When assessing these perceptions in secondary school students, the authors of the original work [26] reported a consistency value of 0.95 with >0.70 for each of the four dimensions. 

### 2.3. Procedure

The questionnaire was created using the Google Forms tool and included the CE scale along with sociodemographic inquiries. The use of an electronic questionnaire was chosen because it facilitated distribution, saved time, and allowed for the storage of all responses in a single database, which increased the return rate.

First, all of the schools that offered primary education were chosen from the directory of educational institutions maintained by the Department of Education and Employment of the Regional Government of Extremadura (https://ciudadano.gobex.es/ciudadanoportlet/printpdf/pdf?typepdf=3443&idDirectorio=775, accessed on 4 March 2022). After that, an email describing the study’s goals and containing an informed parental permission form was sent to all of the school directors. If the schools decided to cooperate together, the students would then need parental agreement in order to participate in the study. The research team was informed at the end of the email that in the event that participants wanted to take part in the study, the physical education teacher was in charge of gathering signed parental informed consent forms and notifying them of the day on which they could attend the classes in person to complete the questionnaire with the children, who were at the time in the fifth or sixth grade of primary school.

Before the questionnaire began, a member of the study team and the center’s physical education teacher read each item and checked to see if the participants had indicated that they had comprehended all the questions. The surveys were filled out on tablets that belonged to the research team and were set up for this purpose to prevent technical problems. The average response time was 10 min, and all data were collected anonymously. Data were gathered between April and May 2022. Students in the fifth and sixth grades responded at rates of 4.2% and 5.2%, respectively. Furthermore, because the study team and the physical education teacher had previously given the questionnaire to the children, the legitimate response rate was 100%.

### 2.4. Statistical Analysis

FACTOR v.10.10.02 (Rovira I Virgili University: Tarragona, Spain), a free statistical program, was used to conduct the exploratory analyses (EFAs), which took into account the ordinal nature of the data acquired using a 5-point Likert scale. While assuming a correlation between dimensions, the factor extraction was carried out using a robust unweighted least squares (RULS) method with Promin rotation [27]. The characteristics of the data were determined using a polychoric correlation matrix, and the correct number of dimensions was established using a parallel analysis [28]. Once the number of dimensions was established, a normalized direct oblimin was selected as the rotation technique to define the factor simplicity and structure. The Kaiser–Meyer–Olkin (KMO) test and Bartlett’s test of sphericity were used as sampling adequacy measures [29].

The AMOS v.26.0.0 software program (IBM Corporation, Wexford, PA, USA) was then used to conduct the confirmatory factor analysis. The items with crossloads greater than 0.40, communalities lower than 0.30, and loads lower than 0.60 were eliminated [30]. The model’s goodness of fit was assessed using the following indicators [31]: the non-normed fit index (NFI); the comparative fit index (CFI); the root mean square error of approximation (RMSEA); the root mean square of residuals (RMSR); the chi-squared probability (*p* > 0.05); and the chi-square per degree of freedom ratio (CMIN/DF). The final configuration of the questionnaire was assessed using the Cronbach’s alpha coefficient and McDonald’s omega reliability metrics [32]. 

## 3. Results

By utilizing a RULS technique with Promin rotation in the first half of the sample, three components related to the explained variance based on eigenvalues and the validity of anticipated a posteriori (EAP) scores [33] were established. In addition, item 15 was deleted prior to the EFAs because its score for the normed measure of sample adequacy (MSA) was less than 0.50 [34]. The sample adequacy indexes produced positive results (Bartlett’s test = 3943.2, df = 496, *p* = 0.000, and KMO test = 0.91595), which led to the execution of the EFAs (the polychoric correlation matrix can be found in Appendix A). A normalized direct oblimin rotation method was chosen once the number of dimensions was specified because the level of kurtosis (kurtosis = 45.034; *p* = 0.000) called for nonparametric methods. The rotated loading matrix for 31 items and three factors is shown in Table 2.

Following the EFA, item 14 was eliminated because its loading was split between two dimensions (Factor 2 (0.366) and Factor 3 (0.317)), which increased the odds of errors in subsequent analyses. The structure and factor loadings of each item are shown in Table 3 (the Spanish version can be found in Appendix B). Three associated dimensions composed the factorial solution.

Similarly, all dimensions showed correlations between them because the values of the inter-factor correlation matrix exceeded the threshold of 0.3 (Table 4).

**Figure 1:**
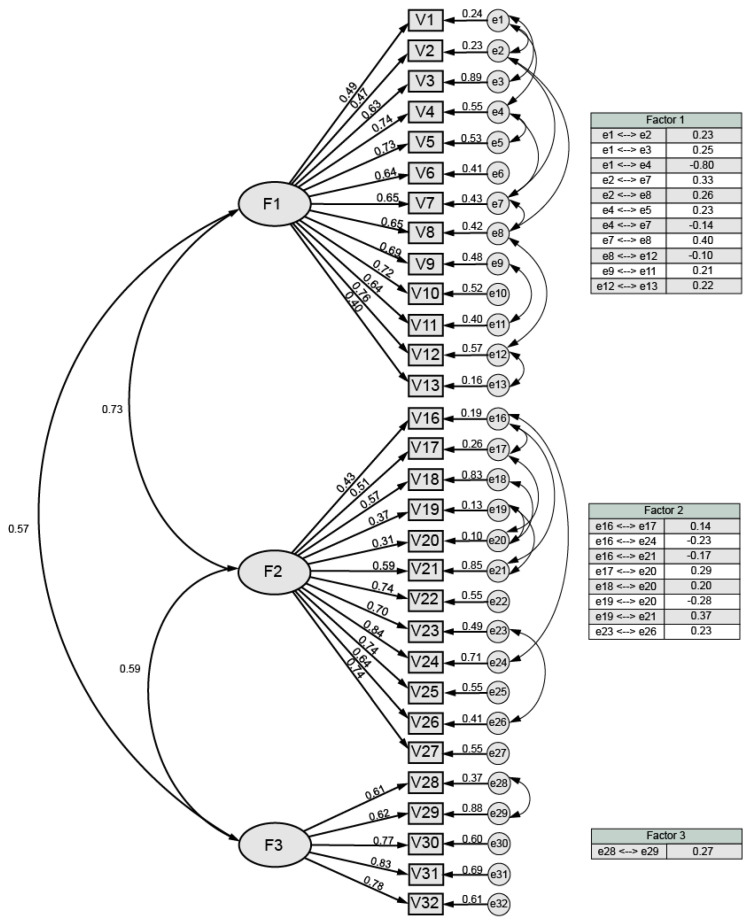
Factorial model of the instrument.

Following the definition of the questionnaire’s structure, the CFA was conducted with the remaining half of the sample to create a definitive model (Figure 1).

Figure 1 depicts the questionnaire’s final structure, which consisted of 30 items divided into three factors. The figure displays the following values (from left to right): correlation between factors, standardized regression weights, squared multiple correlations of each variable, and correlations between exogenous variables (tables).

The goodness-of-fit indices for the instrument after the CFAs, which are displayed in Table 5, each demonstrated a strong fit between the data and the model [36]. Due to the nonsignificant values, the chi-squared probability was very considerable. Additionally, the RMSEA was within the permitted range (0.010–0.050), and the RMSR (at less than 0.08) qualified as accurate. The CMIN/DF index also exhibited excellent values given that it had to be less than 2 to represent an acceptable model fit. NNFI and CFI values greater than 0.9 demonstrated a good fit to the model.

Table 6 shows the reliability indices for the questionnaire dimensions using Cronbach’s alpha, McDonald’s omega, and the explained variance of each factor.

For each of the factors, the Cronbach’s alpha and McDonald’s omega scores were satisfactory because they were higher than 0.7 [37]. The explained variance was the proportion of the variance in the responses that was not attributable to hazard but was instead assigned to each of the model’s dimensions (residual values).

## 4. Discussion

The main objective of the present research was to evaluate the psychometric properties as well as the validity and internal reliability issues of a questionnaire aimed at analyzing the attitudes toward body language of students in the last cycle of primary school (fifth and sixth grades) in the Autonomous Community of Extremadura (Spain). The findings revealed a factor structure composed of three related dimensions and 30 items with excellent goodness-of-fit indices. Moreover, great levels of consistency were shown by the Cronbach’s alpha and McDonald’s omega values. The original questionnaire composed of 32 items divided into four factors was validated for its application in physical education classes at the secondary school level [26]. Finally, our research showed good and exceptional reliability values [38] that were consistent with those provided by previous authors [26].

Instruments such as the one used in this study will allow educational agents at all levels to generate and implement different actions aimed at improving students’ attitudes toward the content of CE [39]. The attitudes of both students and teachers are the main barrier to or facilitator of any educational content [40,41] because these attitudes have emotional, cognitive, and behavioral components and rely more on one than the others [26]. Therefore, the school should be a context in which students’ good attitudes are promoted because such attitudes are not innate but are acquired over time [42]. 

Regarding the evaluation of CE content by students, there were few examples found in the literature that explored this issue, which was one of the main motivations for this study. Vlasic et al. [43] found a positive improvement in student attitudes by providing a specific dance program to Croatian students who were not very familiar with dance. These results were supported by research conducted by Micallef [44] on Maltese secondary school pupils, who reported improvements in rhythm, self-confidence, interaction with others, and knowledge and mastery of their bodies. Conversely, Salz [45] found in an investigation of secondary and high school students’ attitudes toward PE that these were more positive in those contents that students perceived as useful and applicable to their lives, so making students understand how this content extrapolates to everyday life is an essential issue. Similarly, Zeng and coworkers [46] found that the aspects of PE that were most emphasized by students were fun, social relationships, and application they saw in the content outside of school. In this sense, it is believed that the simple features that social networks [47] have (e.g., TikTok) can be leveraged if brought into the context of the subject of body expression in which movement, music, creativity, and rhythm are the key elements [48]. Social networking in secondary education can improve student participation and engagement, unite students, foster a sense of community, provide a learner-centered approach, increase student participation and interaction, stimulate creativity, and improve academic outcomes [49,50]. 

The point of view of students regarding enjoyment of or preference for CE content is particularly important because some subject areas (such as those connected to the block of CE) typically result in students acting in a dismissive, uninterested, and demotivated manner [51]. However, TikTok provides several options for developing a more motivating and interesting learning environment to grab students’ attention [48]. In this section, gender seems to be an influential variable in the activities that compose this content, with girls valuing the importance of CE more highly [41] and showing a greater preference for it, while boys preferred competitive activities [52]. Likewise, students’ perception of their motor competence during CE activities is associated with liking or preference for the content and is typically very low in both genders [53]. Therefore, Holt et al. [54] emphasized the importance of positively influencing students’ motivational processes with respect to CE so that they can become uninhibited and enjoy the activities proposed in this content. Moreover, Fraile-García and collaborators [55] found that students with better academic performance were those who presented an intrinsic motivation oriented toward enjoyment and fun.

Finally, teacher’s attitudes are very important because they influence the way the content is taught and how well or poorly it is received by students [56]. In fact, multiple research studies highlighted the role of the teacher and the content they taught as two of the factors that most influenced schoolchildren’s attitudes because the creation of positive experiences in the classroom depended on the combination of both [26,41]. In a study conducted by Ochoa [57], the participants indicated that the qualities possessed by the teachers that had positively affected their evaluation of CE were knowledge, experience, and conviction together with an emphasis that the teachers themselves placed on the benefits of this practice. Studies by various authors emphasized the importance of the PE teacher in fostering students’ positive attitudes, motivation, and enjoyment regarding the subject [58,59]. In addition, Rady and Schmidt [60] asserted that positive learning environments created by teachers impacted students’ attitudes and learning and that a lack of knowledge in certain areas of PE (such as CE and other artistic content) negatively affected students’ valuing of such content [41]. Teachers should also be aware of the hidden curriculum present in the educational environment and its influence on the formation of positive or negative attitudes toward CE as reported by previous authors [61]. 

### Limitations and Future Lines

The present study had several limitations. The sample was not representative of the entire primary education level because it included only students in the last cycle of primary school (10–12 years of age) without considering the other two cycles. Likewise, it was only carried out in the Community of Extremadura, so sociocultural variables may have influenced the results, in addition to the fact that the participants were selected by means of a convenience sampling method. Secondly, this initial approach to the questionnaire had some disadvantages such as a much smaller sample than that of the present study and the participation of schools from three different autonomous com-munities, in which the contents of physical education may have varied. In addition, our solution grouped the items into four factors despite the fact that two of them (“Preference” and “Pleasure”) had a similar and dependent analysis approach, so the present statistical procedure combined them into a single dimension. It should also be taken into account that keeping item 15 (which does not measure the same domain as the rest of the items of the scale) or item 14 (which had crossloadings between two of the items that composed the questionnaire) were some of the drawbacks that also were present. Thirdly, there were few examples in the literature that studied the evaluation of CE contents by students, which was one of the main motivations for this study. In future areas of research, it would be interesting to extend the sample throughout the Spanish region to gather as much information as possible about CE in primary school students. Likewise, it would be interesting to explore variables that the literature indicated as conditioning factors of the attitudes toward CE (such as gender, previous experience, or age) in order to assess the current state of Spanish primary schoolchildren.

## 5. Conclusions

A questionnaire used to gauge potential student attitudes on CE was analyzed in the current study to determine its validity and reliability. Our results showed that a solution with 30 items and three dimensions had good and outstanding reliability ratings as well as consistent goodness-of-fit indicators. Therefore, this instrument is suitable for administration in the educational environment for both training and research purposes because it is a tool that is easy to use, fast, and ensures high rates of return from students. A positive attitude on the part of students toward CE content is essential for health, social, and psychological benefits as well as for high academic achievement. It is critical to emphasize that contemporary students have diverse routines, interests, and even reasons for their leisure time. In order to tailor educational techniques to their tastes and enable them to discover and appreciate the benefits of CE in their life in a playful manner, it is important to be aware of the aforementioned factors. Moreover, in this sense, it was shown that involving social networks in the teaching–learning process can make this process more motivating for students and one in which they want to participate by enjoying, learning, and exercising at the same time. Therefore, it is recommended that educators propose and implement tasks oriented to the enjoyment and fun of students to positively influence their attitudes. Likewise, teacher attitudes regarding the design of tasks and the teaching–learning process seem to be decisive because they have an intimate relationship with the formation of student attitudes. It should also be taken into account that the school in general should be a context in which good attitudes of students are promoted because such attitudes are not innate but are acquired over time.

## Figures and Tables

**Table 1:** Attributes of the sample (N = 709).

Variables	Categories	N	%
Gender	Male	371	52.3
	Female	338	47.7
Age	10 years	160	22.6
	11 years	474	66.9
	12 years	75	10.6
Center environment	Urban	341	48.1
	Rural	368	51.9
Type of center	Public	667	94.1
	Private	42	5.9
Course	Fifth grade of primary school	318	44.9
	Sixth grade of primary school	391	55.1
Province	Cáceres	506	71.4
	Badajoz	203	28.6

N: number; %: percentage.

**Table 2:** Rotated loading matrix.

Item	Factor 1	Factor 2	Factor 3
1. Corporal Expression is useful for my training.	−0.135	−0.049	0.613
2. Corporal Expression allows to express my feelings.	−0.088	−0.079	0.777
3. The learning received in Corporal Expression is necessary and important.	0.129	0.057	0.650
4. Corporal Expression classes improve the mood.	0.061	0.206	0.605
5. Corporal Expression helps to know oneself better, to relate to others and to be creative.	−0.086	0.091	0.798
6. Corporal Expression contributes to global education.	−0.019	−0.005	0.731
7. Corporal Expression is good for socialization.	0.047	−0.031	0.709
8. Corporal Expression is a good social experience and gives you opportunities to get to know your peers in a deeper way.	−0.012	0.068	0.790
9. In the Corporal Expression classes a very positive environment is created.	0.148	0.083	0.634
10. Corporal Expression provides important relief from accumulated stress.	0.024	0.149	0.589
11. Corporal Expression also improves overall health and not only physical fitness activities.	0.107	0.126	0.628
12. The activities taught in Corporal Expression seem important to me.	0.227	0.208	0.529
13. What is learned in Corporal Expression is useless.	0.261	−0.064	0.432
14. I like Corporal Expression because it works on aesthetics and social relations.	0.177	0.366	0.317
15. I don’t like Corporal Expression because it doesn’t have as much risk or as many challenges as sports.	Deleted		
16. I find Coporal Expression interesting because it is not competitive.	0.145	0.402	0.109
17. I prefer Corporal Expression to other contents.	−0.112	0.678	−0.102
18. I prefer Corporal Expression because students interact with their peers more than when doing other motor skills contents.	−0.024	0.618	0.154
19. Corporal Expression is not as fun as other content.	0.098	0.391	0.155
20. Corporal Expression is more important than the rest of the contents.	−0.187	0.680	−0.062
21. If doing Corporal Expression in the classes were optional, I would choose to do it.	0.038	0.646	0.126
22. When I have taken Corporal Expression classes I have liked it because it is something different from what is normally taught.	0.117	0.629	0.158
23. When I have taken Corporal Expression classes I have liked it because it is cooperative.	0.289	0.442	0.212
24. When I have taken Corporal Expression classes I have enjoyed the time I have spent doing these activities.	0.188	0.633	0.156
25. When I have taken Corporal Expression classes, I have liked them because they include artistic activities.	0.101	0.557	0.160
26. When I have taken Corporal Expression classes, I have liked them because they involve more games.	0.289	0.563	−0.086
27. When I have taken Corporal Expression classes, I have always wanted more.	0.235	0.585	0.114
28. The teacher values Corporal Expression.	0.707	0.030	−0.021
29. The teacher provides opportunities for the development of expressive skills.	0.671	0.042	0.021
30. The teacher tries to make the Corporal Expression sessions fun.	0.852	−0.035	−0.043
31. My PE teacher makes the Corporal Expression class useful for me.	0.812	−0.006	0.018
32. I feel that my PE teacher makes learning in Corporal Expression valuable to me.	0.804	0.028	0.064

Note: these items are a literal translation into English for ease of reading and not a cross-cultural adaptation into English. Adapted with permission from [35].

**Table 3:** Rotated factor loadings and factor solutions.

Item	Factor 1	Factor 2	Factor 3
1. Corporal Expression is useful for my training.			0.613
2. Corporal Expression allows to express my feelings.			0.777
3. The learning received in Corporal Expression is necessary and important.			0.650
4. Corporal Expression classes improve the mood.			0.605
5. Corporal Expression helps to know oneself better, to relate to others and to be creative.			0.798
6. Corporal Expression contributes to global education.			0.731
7. Corporal Expression is good for socialization.			0.709
8. Corporal Expression is a good social experience and gives you opportunities to get to know your peers in a deeper way.			0.790
9. In the Corporal Expression classes a very positive environment is created.			0.634
10. Corporal Expression provides important relief from accumulated stress.			0.589
11. Corporal Expression also improves overall health and not only physical fitness activities.			0.628
12. The activities taught in Corporal Expression seem important to me.			0.529
13. What is learned in Corporal Expression is useless.			0.432
14. I like Corporal Expression because it works on aesthetics and social relations.	Deleted		
15. I don’t like Corporal Expression because it doesn’t have as much risk or as many challenges as sports.	Deleted		
16. I find Coporal Expression interesting because it is not competitive.		0.402	
17. I prefer Corporal Expression to other contents.		0.678	
18. I prefer Corporal Expression because students interact with their peers more than when doing other motor skills contents.		0.618	
19. Corporal Expression is not as fun as other content.		0.391	
20. Corporal Expression is more important than the rest of the contents.		0.680	
21. If doing Corporal Expression in the classes were optional, I would choose to do it.		0.646	
22. When I have taken Corporal Expression classes I have liked it because it is something different from what is normally taught.		0.629	
23. When I have taken Corporal Expression classes I have liked it because it is cooperative.		0.442	
24. When I have taken Corporal Expression classes I have enjoyed the time I have spent doing these activities.		0.633	
25. When I have taken Corporal Expression classes, I have liked them because they include artistic activities.		0.557	
26. When I have taken Corporal Expression classes, I have liked them because they involve more games.		0.563	
27. When I have taken Corporal Expression classes, I have always wanted more.		0.585	
28. The teacher values Corporal Expression.	0.707		
29. The teacher provides opportunities for the development of expressive skills.	0.671		
30. The teacher tries to make the Corporal Expression sessions fun.	0.852		
31. My PE teacher makes the Corporal Expression class useful for me.	0.812		
32. I feel that my PE teacher makes learning in Corporal Expression valuable to me.	0.804		

Note: adapted with permission from [35].

**Table 4:** Inter-factor correlation matrix.

Factors	Factor 1	Factor 2	Factor 3
Factor 1	1.000		
Factor 2	0.362	1.000	
Factor 3	0.417	0.508	1.000

**Table 5:** Questionnaire goodness-of-fit indices.

Indices	Value
NNFI	0.901
CFI	0.931
RMSEA	0.047
RMSR	0.072
Ρ (*χ*^2^)	0.773
CMIN/DF	1.889

**Table 6:** Goodness of fit indices.

Parameters	Factor 1	Factor 2	Factor 3
Cronbach’s alpha	0.905	0.878	0.855
McDonald’s omega	0.907	0.879	0.855
Explained variance	4.030	4.946	6.663

## Data Availability

The datasets are available through the corresponding author upon reasonable request.

## Appendix A

**Table A1:** Polychoric correlation matrix extracted from the exploratory analysis.

Item	1	2	3	4	5	6	7	8	9	10	11	12	13	16	17	18	19	20	21	22	23	24	25	26	27	28	29	30	31	32
1	1.00																													
2	0.604	1.00																												
3	0.418	0.580	1.00																											
4	0.323	0.432	0.561	1.00																										
5	0.396	0.536	0.578	0.683	1.00																									
6	0.439	0.481	0.591	0.572	0.643	1.00																								
7	0.427	0.504	0.459	0.437	0.595	0.546	1.00																							
8	0.381	0.570	0.541	0.572	0.661	0.563	0.661	1.00																						
9	0.352	0.471	0.510	0.613	0.585	0.442	0.512	0.675	1.00																					
10	0.267	0.428	0.433	0.566	0.524	0.444	0.402	0.632	0.622	1.00																				
11	0.325	0.444	0.536	0.593	0.569	0.501	0.510	0.616	0.630	0.640	1.00																			
12	0.335	0.403	0.609	0.565	0.584	0.550	0.586	0.622	0.542	0.509	0.548	1.00																		
13	0.134	0.288	0.466	0.419	0.377	0.317	0.342	0.391	0.367	0.343	0.433	0.538	1.00																	
16	0.198	0.248	0.256	0.299	0.328	0.192	0.321	0.358	0.348	0.273	0.344	0.354	0.253	1.00																
17	0.183	0.062	0.184	0.239	0.217	0.157	0.150	0.200	0.179	0.134	0.223	0.253	−0.036	0.306	1.00															
18	0.249	0.256	0.423	0.445	0.435	0.378	0.345	0.446	0.393	0.313	0.356	0.448	0.126	0.434	0.490	1.00														
19	0.087	0.239	0.318	0.358	0.299	0.237	0.253	0.337	0.370	0.326	0.391	0.358	0.373	0.341	0.286	0.374	1.00													
20	0.130	0.119	0.207	0.235	0.219	0.225	0.138	0.247	0.154	0.193	0.130	0.268	−0.045	0.246	0.519	0.472	0.117	1.00												
21	0.197	0.323	0.386	0.436	0.399	0.298	0.305	0.414	0.401	0.375	0.399	0.497	0.289	0.412	0.419	0.475	0.541	0.332	1.00											
22	0.163	0.319	0.396	0.489	0.487	0.337	0.382	0.487	0.433	0.417	0.471	0.537	0.354	0.465	0.351	0.479	0.440	0.393	0.636	1.00										
23	0.230	0.330	0.492	0.463	0.456	0.373	0.425	0.459	0.536	0.488	0.491	0.526	0.339	0.329	0.292	0.466	0.362	0.299	0.535	0.581	1.00									
24	0.289	0.355	0.449	0.555	0.405	0.388	0.379	0.440	0.472	0.434	0.529	0.546	0.390	0.434	0.392	0.457	0.461	0.368	0.606	0.722	0.632	1.00								
25	0.185	0.323	0.452	0.443	0.408	0.374	0.299	0.377	0.376	0.438	0.457	0.485	0.287	0.348	0.282	0.431	0.377	0.369	0.456	0.564	0.475	0.625	1.00							
26	0.130	0.148	0.272	0.317	0.242	0.234	0.244	0.300	0.327	0.336	0.327	0.403	0.166	0.333	0.240	0.465	0.297	0.342	0.419	0.438	0.579	0.496	0.532	1.00						
27	0.160	0.311	0.443	0.573	0.465	0.319	0.324	0.389	0.458	0.485	0.471	0.553	0.276	0.364	0.352	0.468	0.367	0.319	0.540	0.555	0.567	0.692	0.622	0.587	1.00					
28	0.088	0.193	0.318	0.275	0.175	0.207	0.193	0.254	0.263	0.208	0.258	0.392	0.293	0.251	0.059	0.247	0.242	0.014	0.301	0.269	0.370	0.344	0.304	0.311	0.337	1.00				
29	0.155	0.205	0.337	0.280	0.296	0.275	0.280	0.255	0.324	0.141	0.280	0.334	0.212	0.282	0.118	0.299	0.163	0.091	0.280	0.289	0.411	0.330	0.225	0.334	0.376	0.611	1.00			
30	0.065	0.115	0.295	0.274	0.178	0.235	0.238	0.225	0.363	0.254	0.320	0.367	0.323	0.254	0.050	0.191	0.179	−0.016	0.196	0.339	0.397	0.363	0.283	0.380	0.367	0.629	0.606	1.00		
31	0.128	0.202	0.350	0.320	0.232	0.243	0.315	0.296	0.382	0.236	0.313	0.446	0.326	0.277	0.173	0.199	0.254	0.077	0.227	0.307	0.429	0.388	0.286	0.365	0.386	0.522	0.557	0.633	1.00	
32	0.105	0.197	0.413	0.388	0.284	0.277	0.329	0.378	0.406	0.337	0.403	0.507	0.331	0.332	0.150	0.256	0.280	0.117	0.267	0.388	0.480	0.421	0.295	0.372	0.440	0.534	0.562	0.645	0.853	1.00

## Appendix B

**Table A2:** Cuestionario para la evaluación de las actitudes del alumnado hacia el contenido de expresión corporal.

1. La expresión corporal es útil para mi formación
2. La expresión corporal permite expresar mis sentimientos
3. El aprendizaje recibido en la Expresión Corporal es necesario e importante
4. Las clases de Expresión Corporal mejoran el estado de ánimo.
5. La Expresión Corporal ayuda a conocerse mejor, a relacionarse con los demás y a ser creativo.
6. La Expresión Corporal contribuye a una educación global.
7. La Expresión Corporal es buena para la socialización.
8. La Expresión Corporal es una buena experiencia social y te da la oportunidad de conocer a tus compañeros de una manera más profunda.
9. En las clases de Expresión Corporal se crea un ambiente muy positivo.
10. La Expresión Corporal proporciona un alivio importante del estrés acumulado.
11. La Expresión Corporal también mejora la salud en general y no sólo las actividades de acondicionamiento físico.
12. Las actividades que se enseñan en Expresión Corporal me parecen importantes.
13. Lo que se aprende en Expresión Corporal no sirve para nada.
14. Me gusta la Expresión Corporal porque trabaja la estética y las relaciones sociales.
15. No me gusta la Expresión Corporal porque no tiene tanto riesgo ni tantos retos como los deportes.
16. La Expresión Corporal me parece interesante porque no es competitiva.
17. Prefiero la Expresión Corporal a otros contenidos.
18. Prefiero la Expresión Corporal porque los alumnos interactúan más con sus compañeros que cuando realizan otros contenidos de habilidades motrices.
19. La Expresión Corporal no es tan divertida como otros contenidos.
20. La Expresión Corporal es más importante que el resto de contenidos.
21. Si hacer Expresión Corporal en las clases fuera opcional, elegiría hacerla.
22. Cuando he tomado clases de Expresión Corporal me ha gustado porque es algo diferente a lo que normalmente se enseña.
23. Cuando he tomado clases de Expresión Corporal me ha gustado porque es cooperativo.
24. Cuando he tomado clases de Expresión Corporal he disfrutado el tiempo que he pasado haciendo estas actividades.
25. Cuando he tomado clases de Expresión Corporal me han gustado porque incluyen actividades artísticas.
26. Cuando he tomado clases de Expresión Corporal, me han gustado porque incluyen más juegos.
27. Cuando he tomado clases de Expresión Corporal, siempre me he quedado con ganas de más.
28. El profesor valora la Expresión Corporal.
29. El profesor proporciona oportunidades para el desarrollo de las habilidades expresivas.
30. El profesor intenta que las sesiones de Expresión Corporal sean divertidas.
31. Mi profesor de Educación Física hace que la clase de Expresión Corporal sea útil para mí.
32. Siento que mi profesor de Educación Física hace que el aprendizaje en Expresión Corporal sea valioso para mí.

Note: adapted with permission from [35].
